# Human myeloid differentiation by BMP4 signaling through the VDR pathway in acute myeloid leukemia

**DOI:** 10.1038/s41420-024-02090-4

**Published:** 2024-07-16

**Authors:** Florence Zylbersztejn, Iryna Byelinska, Sandrine Jeanpierre, Léa Barral, Kevin Geistlich, Mario Flores-Violante, Thibault Voeltzel, Etienne Paubelle, Mael Heiblig, Vincent Alcazer, Gregoire Le Meur, Gaelle Fossard, Amine Belhabri, Ivan Cruz-Moura, Olivier Hermine, Sylvain Lefort, Véronique Maguer-Satta

**Affiliations:** 1 CNRS UMR5286, Centre de Recherche en Cancérologie de Lyon, 69000 Lyon, France; 2 Inserm U1052, Centre de Recherche en Cancérologie de Lyon, 69000 Lyon, France; 3 Department of Cancer Initiation and Tumor Cell Identity, Lyon, France; 4 Université Claude Bernard Lyon 1, CRCL, 69000 Lyon, France; 5 Necker Hospital, Imagine Institute, Inserm U116 CNRS ERL 8654, 75015 Paris, France; 6 Department of Clinical Medicine, Educational and Scientific Center “Institute of Biology and Medicine”, Taras Shevchenko National University of Kyiv, Kyiv, Ukraine; 7 Centre Léon Bérard, 69000 Lyon, France; 8 Hospices Civils de Lyon, Hematology Department, Centre Hospitalier Lyon Sud, 69495 Pierre Bénite, France

**Keywords:** Cancer stem cells, Diseases

**Fig. 1:**
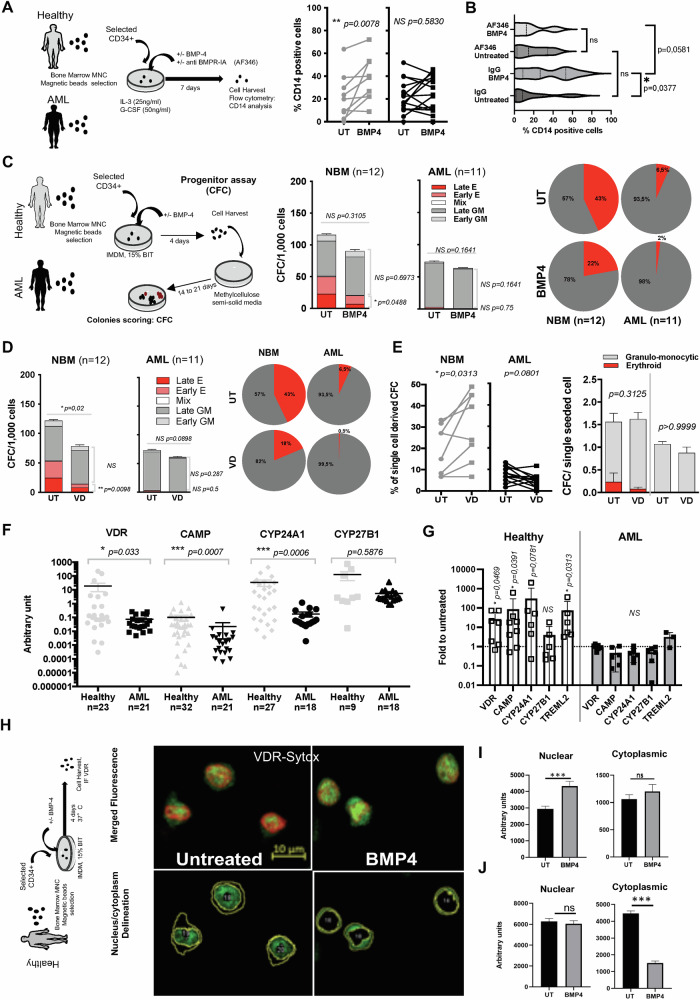
Response of bone marrow immature cells to BMP4 and VD mediated-myeloid progenitors’ differentiation is lost in AML. **A** Experimental protocol for differentiation analysis of normal and leukemic samples. CD34^+^ from healthy and AML samples were cultured at 0.4 M/mL in serum-free medium in the presence of IL-3 (10 ng/mL), G-CSF (50 ng/mL), and BMP4 treatment for 7 days. Cell membrane analysis of CD14^+^ was performed by flow cytometry, and data represent the percentage of positive cells. Healthy CD34^+^ samples are presented in gray (*n* = 8) and AML samples in black (*n* = 15). Wilcoxon matched-pairs signed rank test **B** CD34^+^ from healthy samples were also treated with or without BMPR1A blocking antibody (AF346 R&D System), and data are presented as violin plot of *n* = 5 independent experiments using CD34^+^ isolated from different healthy donors. **C** The progenitor content of treated cells was analyzed using the clonogenic CFC assay. We scored them as early or late erythroid-E and early or late granulo-monocytic-GM colonies. Results are expressed as the number of CFC for 1000 cells and represent the mean value ± SEM *n* = 14. **D** CD34^+^ from healthy and AML samples were cultured for 4 days at 0.4 M/mL in serum-free medium in the presence of VD (300 nM) treatment. The progenitor content of treated cells was analyzed using the clonogenic CFC assay. We scored them as early or late erythroid-E and as early or late granulo-monocytic-GM. Results are expressed as the number of CFC for 1000 cells and represent the mean value ± SEM of 12 or 11 experiments for healthy donors and AML patients, respectively. **E** Frequency of single cell-derived CFC and absolute number of colonies per CFC obtained and type. Results are expressed as the percentage of wells that gave rise to colonies and represent the mean value ± SEM of five experiments for healthy donors and 7 AML patients. UT Untreated, NBM Normal Bone Marrow, CFC Colony-Forming Capacity. **F** Transcript levels in bone marrow CD34^+^ healthy (gray) and AML samples (black) were evaluated by RT-PCR using primers (Table  S2). **G** After 4 days of BMP4 exposure of CD34^+^ isolated from NBM or AML samples transcript levels of TREML2, VDR, and its target genes were evaluated by RT-PCR. **H** VDR receptor (green fluorescence revealed by green staining with secondary anti-rabbit AlexaFluoro-488 conjugated antibody) confocal microscopy images of healthy donor bone marrow-derived CD34+ cells following BMP4 treatment (20 ng/mL during 4 days). **I** The VDR receptor distribution in the cytoplasmic and nuclear region of CD34+ bone marrow-derived cells of a healthy donor, confocal microscopy images of VDR receptor and analysis of stain intensity in cytoplasmic and nuclear region (Sytox deep red staining of nuclei). **J** KG1A confocal microscopy images of VDR receptor, green fluorescence (Sytox deep red staining of nuclei), and analysis of stain intensity in the cytoplasmic and nuclear region of cells after BMP4 treatment (20 ng/mL during 4 days). Graphpad Prism analysis and paired *t*-test were performed unless specified.

**To the Editor**,

Acute myeloid leukemia (AML) is a heterogeneous disease with the accumulation of cytogenetic or genetic aberrations leading to the overgrowth of malignant cells and in which the first cancer stem cells (SC) have been identified (leukemic stem cells—LSC) [1]. The dynamic crosstalk between LSC and their bone marrow (BM) microenvironment is crucial for controlling leukemic progression and response to therapy [2]. Among the main pathways that participate in this dialog, the Bone Morphogenetic Proteins (BMP) signaling constitutes a major bi-directional actor as BMP molecules govern SC regulation in developmental stages and many adult tissues [3]. Intrinsic and extrinsic alterations of BMP signaling have been identified at different stages of myeloid leukemia, including initiation, expansion, persistence, and resistance of immature cells [3–7]. We unveiled a signaling cascade involving the binding of BMP4 to BMPR1A that drives ΔNp73 and NANOG expression to imprint immature-like properties and which is predictive in the diagnosis of AML patients at risk of relapse [5]. Bioinformatics analyses also identified BMP4 as one of the key hub genes and pathways in AML [8]. In normal human hematopoiesis, we showed that BMP2 and BMP4 regulate HSC maintenance [9], commitment toward erythroid [10], and megakaryocytic lineages [9]. Regarding existing data on other lineage regulation, the lack of extensive data on myeloid lineage regulation by BMP4 and the fact that we recurrently document an increase in BMP4 in myeloid leukemia at the time of diagnosis [5] or at relapse [7], we wondered if BMP4 could contribute to myeloid cell differentiation. We isolated CD34^+^ cells from BM samples collected from healthy donors (NBM) or AML patients at diagnosis (Table S1). Cells were treated with BMP4 (20 ng/mL) as described (Fig. 1A). The induction of myeloid differentiation was quantified by CD14 cell surface expression (a marker of monocytic cells). We observed a significant induction of CD14-expression in healthy (Fig. 1A, left panel) but not AML (Fig. 1A, right panel) BMP4-treated CD34^+^ cells. Using a BMPR1A blocking antibody (AF346) [5] prevented the BMP4-induced CD14 expression in healthy CD34^+^ cells (Fig. 1B), confirming that BMP4 signaling is directly involved in monocytic differentiation. In the NBM progenitors compartment, exposure to BMP4 led to a slight decrease in total colonies (CFC) (Fig. 1C, left panel) but induced a strong reduction of erythroid progenitors (early-BFU-E and late-CFU-E) in healthy BMP4-treated cells (2 folds)(Fig. 1C, pie charts). Even if erythroid progenitors’ frequency in AML BM samples is very low, BMP4 treatment induces a further 3-fold decrease. We performed functional assays (Long Term Culture-Initiating Cell; LTC-IC) to assess the effect of BMP4 on NBM and AML immature CD34^+^ cells. Their capacity to generate progenitors after 5 weeks of co-culture with the feeder layer was unaltered (Supplementary Fig. 1A), indicating that BMP4 does not foster HSC expansion in these conditions. To evaluate the effect on HSC commitment toward different lineages, we performed single-cell functional analyses on CD34^+^CD38^−^CD90^+^CD123^+^ sorted sub-fraction, highly enriched in HSC. Single sorted cells were treated for 10 days with BMP4 prior to semi-solid medium addition, and progenitors scored after another period of 14–21 days (Supplementary Fig. 1B, left panel). While, as expected, sorted AML LSC fate is biased toward the myeloid lineage, BMP4 neither changes the capacity of HSC to generate colonies (Supplementary Fig. 1B, middle panel), nor to direct their fate toward erythroid or myeloid lineages (Supplementary Fig. 1B, right panel). Therefore, BMP4 controls healthy immature cells by promoting the myeloid progenitor’s compartment expansion at the expense of erythroid progenitors but without instructing HSC commitment. This BMP4 effect on myeloid differentiation is lost in AML LSC.

To gain insight into the mechanism by which BMP4 regulates the myeloid lineage, we evaluated well-established regulatory signaling pathways and chose Vitamin D (VD) signaling and its receptor VDR involved in myeloid differentiation and altered in AML [11]. We exposed 7 days of NBM or AML CD34^+^-sorted cells to VD (300 nM) and observed, as expected, the induction of CD14 surface expression in NBM cells (Supplemental Fig. 1C). This effect was not observed in CD34^+^ AML primary cells confirming that VD induced myeloid differentiation is lost in leukemic cells. As with BMP4-treatment (Fig. 1C), VD-treated CD34^+^ cells decreased the total number of progenitors with a significant drop from 43% to 18% in the erythroid compartment (respectively early and late sub-types)(Fig. 1D pie Chart). Unlike healthy cells, AML CD34^+^ cells displayed a reduced capacity to generate progenitors in CFC assays, which remained unaffected by VD treatment (Fig. 1D left panels). Conversely to BMP4 (Supplementary Fig. 1B), VD treatment enhanced the frequency of colonies generated from healthy single stem cells (Fig. 1E left panel) without significantly affecting their fate between erythroid or myeloid lineages (Fig. 1E right panel). Our data show comparable effects of BMP4 and VD signaling on healthy primary stem cells to promote myeloid progenitor expansion and favor CD14^+^ cells, effects abrogated in AML-sorted LSC.

At the molecular level, we observed a significantly lower expression in AML cells of VDR and of its target genes directly involved in myeloid differentiation (CAMP and CYP24A1 [11]), but not of CYP27B1, involved in T lineage differentiation [12] (Fig. 1F). Based on an AML dataset (GSE76008) [13] we observed a positive correlation between the expression of BMPR1A and of VDR in unsorted cells (Supplementary Fig. 1D), and a lower transcriptional level of VDR and CAMP genes in CD34^+^ compare to CD34^-^ cells from the same patients (Supplementary Fig. 1F), suggesting that the BMP4-induced myeloid regulation could involve VD signaling. Treating CD34^+^ cells with BMP4 (20 ng/mL) for 4 days led to a significant increase in the expression of VDR, its target genes (CAMP, CYP24A1) and TREML2 (also implicated in myeloid differentiation [14]) only in NBM cells, while this up-regulation was not observed in BMP4-treated AML cells (Fig. 1G). VDR is a nuclear receptor that, following its cytoplasmic to nuclear translocation, drives transcriptional activation of factors to induce myeloid differentiation. Confocal analysis confirmed the overall lower level of VDR in CD34^+^ AML cells compared to their normal counterparts (Supplementary Fig. 1G). Following BMP4 treatment, we observed by compartment delineation an increase in VDR nuclear staining in NBM CD34^+^-cells, but no difference in its cytoplasmic localization (Fig. 1H, I). While similar VDR shuttling to the nucleus in response to BMP4 treatment was measured in mature HL60-AML cells (Supplementary Fig. 1H), this was not the case in immature CD34^+^-AML KG1A cells (Fig. 1J). In addition, KG1A cells showed no cytoplasmic VDR staining, confirming a decrease in VDR expression in AML CD34^+^ cells (Fig. 1J). Altogether, our data strongly indicate a loss of VDR regulation by BMP4 in immature AML cells, including a dysregulated VDR shuttling between cellular compartments. Interestingly, it was suggested that AML-associated translocation products block differentiation by interfering with chromatin-modeling but also by sequestering factors such as VDR in the VD-induced differentiation [15]. Our data thus raise the hypothesis of an alteration of LSC control by BMP4 through re-localization of VDR in the absence of transcriptional regulation. This supports the participation of the BMP pathway in blocking cell differentiation through non-genomic VDR activity.

Hence, our data show that in a normal context, BMP4 binding to BMPR1A contributes to myeloid differentiation through VDR pathway activation. In AML, this mechanism is impaired, likely due to a change in VDR localization mediated by BMP4, which likely prevents its nuclear pro-differentiation function. These findings provide insight into the mechanism of differentiation blockade and myeloid expansion of AML LSC, opening new clinical perspectives to restore VD pro-differentiation potential.

### Supplementary information

Zylbersztejn CDD Supplemental data 25-06-2024 R
