# Supplementary material for: Human myeloid differentiation by BMP4 signaling through the VDR pathway in acute myeloid leukemia
Source: Cell Death Discov. 2024 Jul 16;10:325. doi: 10.1038/s41420-024-02090-4 (PMC11252393; doi:10.1038/s41420-024-02090-4)
Supplement: Supplementary file 1 — Zylbersztejn CDD Supplemental data 25-06-2024 R [file 41420_2024_2090_MOESM1_ESM.pdf]

Supplemental Figure and Methods

Zylbersztejn et al supplemental Figure 1

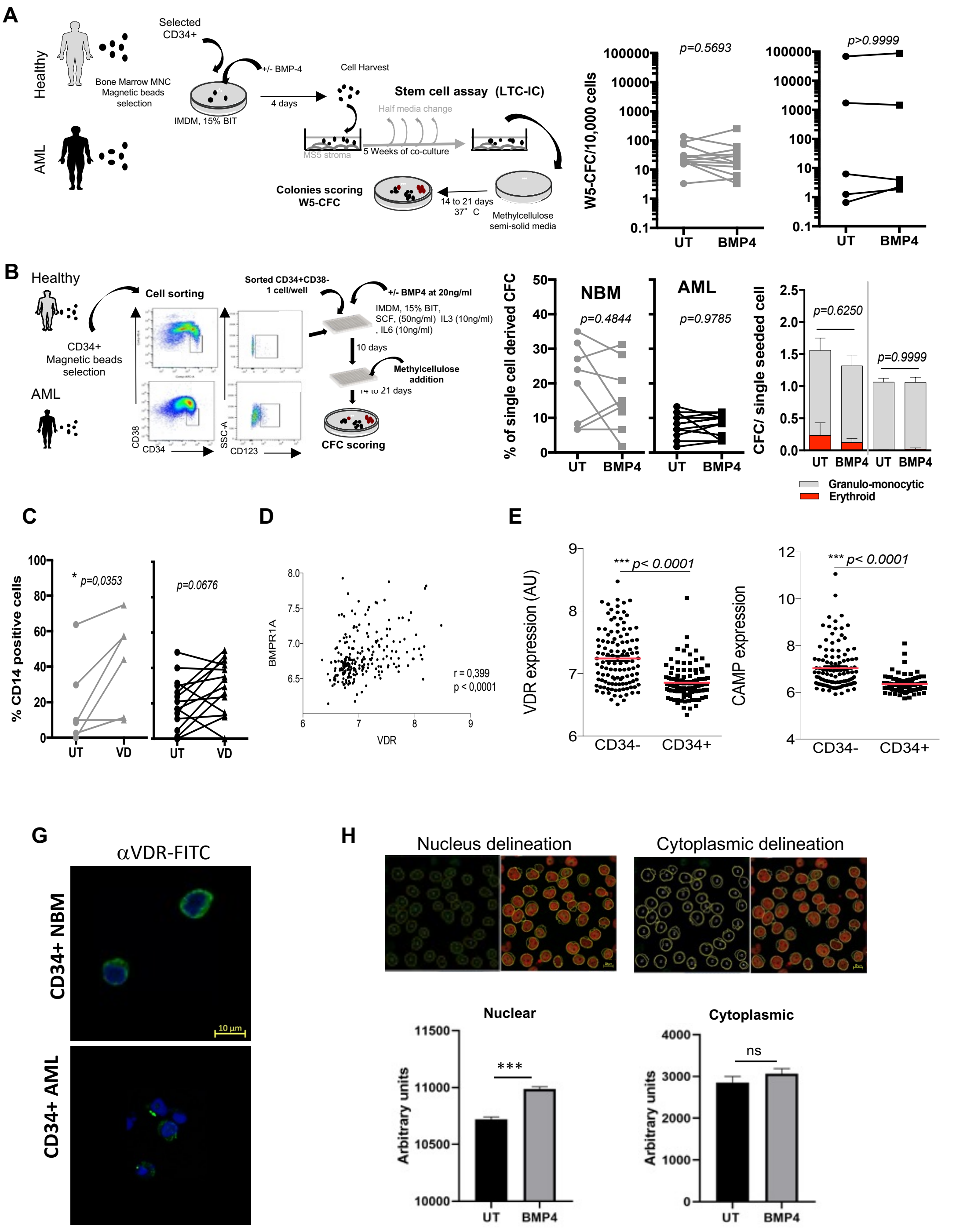

Supplemental Figure 1: (A) Experimental procedure for LTC-IC output and presented as mean +/- SEM of week5-derived CFC colonies for 1,000 input cells. (B) Experimental protocol for functional analysis of normal and leukemic samples at single-cell level. Sorted CD34^{+}/CD38^{-}/CD123^{+} cells from healthy and AML samples were plated at 1 cell/well in serum-free medium in the presence of 20ng/mL BMP4. After 10 days, methylcellulose was added to the wells, and single cell-derived colonies were scored 2 weeks later. Frequency of single cell-derived CFC and absolute number of colonies per CFC obtained and type. Results are expressed as percentage of wells that gave rise to colonies and represent the mean value +/- SEM of 5 experiments for healthy donors and 7 AML patients. (C) CD34+ from healthy and AML samples were cultured at 0.4M/mL in serum-free medium in the presence of IL-3 (10ng/mL) and G-CSF (50ng/mL) and VD (300nM) treatment for 7 days. N=8. Cell membrane analysis of CD14+ was performed by flow cytometry, and data represent the percentage of positive cells. (D) Correlation of BMPR1A and VDR transcript levels in paired CD34^{+} and CD34- cells isolated from AML samples. Data were obtained from the GSE76008 (13) (E) Retrospective analysis of a genebank dataset study (GSE7600820) showing VDR expression in a cohort composed of leukemic CD34+ and CD34- BM samples. (G) CD34+ cells from NBM or AML (DAPI staining of nuclei, blue-fluorescence) (H) HL60 confocal microscopy images of VDR receptor, green fluorescence (Sytox deep red staining of

nuclei) and analysis of stain intensity in cytoplasmic and nuclear region of cells after BMP4 treatment (20ng/mL during 4 days).

Methods

Cells.

Samples were obtained from healthy BM donors for allogeneic transplant or BM from AML patients at diagnosis before treatment. All donors provided written informed consent in accordance with the Declaration of Helsinki. Studies were approved by local ethics committee bylaws. For the BRC of CLB (n°BB-0033-00050) biological material collection and retention activity is declared to the Ministry of Research (DC-2008-99 and AC-2019-3426). Samples were used in the context of patient diagnosis (BB-0033-00050, CRB Centre Léon Bérard, and M1-P1-ST1.1 BRC-HCL; Lyon France. This study was approved by the ethical review board of Centre Léon Bérard. This BRC quality certified according to AFNOR NFS96900 (N° 2009/35884.2) and ISO 9001 (Certification N° 2013/56348.2). CD34 immunomagnetic separation (StemCell Technologies, Vancouver, BC, Canada) reached an average purity of 90%. Primary cells were maintained 4 days in IMDM (Invitrogen) 15% BSA, insulin and transferrin (BIT) (StemCell Technologies, Vancouver, BC, Canada). BMP4 (Sigma-Aldrich) and Vitamin D3 (Calcitriol Desma Pharma) were added in serum free medium respectively at 20ng/ml and 300nM. When used, normal goat IgG control (AB-108-C) and anti-hBMPR1A (AF346) (R&D systems) were added at 4µg/mL. KG1A were purchased from ATCC. Mycoplasma test are performed every 2 months to ensure mycoplasma free culture.

For differentiation assays, cells were cultured at 0.4 10^6 cells/mL in Iscove modified Dulbecco medium (Invitrogen); 15% bovine serum albumin, insulin, and transferrin (Stemcell Technologies); 10 ng/mL interleukin 3 (IL-3) and 50ng/mL G-CSF (Peprotech) for 7 days.

Table S1: AML Patients data

Code Sample	Sex	Age	Diag	% blastes	Anomalies	FAB	ELN2022
LAM243	F	69	Yes	17	NPM1mut	FAB5	Good
LAM244	M	52	Yes	95	MLL rearranged	FAB1	Poor
LAM6	M	23	Yes	90	FLT3-ITD, WT1mut	FAB5	Intermediate
LAM266	M	32	Yes	80	FLT3-ITD, RUNX1 double muté, PTPN11 muté, BCORL1 muté	FAB5	Poor
LAM268	M	37	Yes	55	t(8;21) AML1-ETO	FAB2	Good
LAM269	M	72	Yes	70	Caryotype hyperdiploïde, +8, IDH1	FAB2	Intermediate
LAM271	M	72	Yes	85	NPM1mut, IDH1	FAB2	Intermediate
LAM279	M	28	Yes	95	-Y, t(6;21) (réarrangement RUNX1), FLT3-ITD	FAB5	Intermediate
LAM 224	F	66	Yes	80	FLT3-ITD	FAB1	Intermediate

Functional assays.

Colony forming cell (CFC) and long-term culture-initiating cell (LTC-IC) assays were performed as reported [11]. LTC-IC assay quantify hematopoietic stem cells by their ability to sustain the production of various hematopoietic progenitors (erythroid and myeloid) after a long-term period of co-culture with a stroma feeder and revealed using plating of harvested cell in a semi solid media (methylcellulose, Colonies Forming Cells-CFC assay). LTC-IC number was expressed as W5-CFC/10,000 initial cells. MS5 were used as feeders in LTC-IC assays. Fresh CD34+ cells from healthy or AML patients were harvested and cultured for four days in serum free IMDM (Invitrogen) 15% BSA, insulin and transferrin (BIT) (StemCell Technologies). Vitamin D3 (Calcitriol Desma Pharma) was added at 300nM in the presence or not of BMP4 (20ng/mL) and/or anti-hBMPR1A (4µg/mL) (R&D Systems). Viable cells were counted by trypan blue exclusion.

For the single cell–derived-CFC formation frequency, cells were directly sorted into U-bottom 96-well plates containing serum free IMDM (Invitrogen) 15% BSA, insulin and transferrin (BIT) (StemCell Technologies) supplemented with 10ng/mL of interleukin-3 (IL-3) and interleukine-6 (IL-6), and 50ng/mL of stem cell factor (SCF) during 10 days with Vitamin D3 (300nM) or BMP4 (20ng/mL). Methylcellulose (H4230, StemCell Technologies, Vancouver, BC, Canada) was added and CFC derived-cell counted and classified after 7-14 days.

RNA isolation and analysis

Quantitative RT-PCR was performed using standard protocols (ref). Primary cells were isolated by a Ficoll gradient and total RNA was purified using TRI REAGENT™ (Sigma). For RT-qPCR, cDNA was produced using Superscript II (Invitrogen) and amplified using Sybr-green (Quantifast, Qiagen) and the Real-Time PCR system (Roche). TBP (TATA-binding protein) and HPRT (hypoxanthine-guanine phosphoribosyl transferase) genes were used for normalization. Arbitrary Unit (AU) corresponds to the ratio of expression between samples and a single normal sample used as a reference in each PCR.

Table S2: Primer sequences

Gene	Forward	Reverse
TBP	5′-CACGAACCACGGCACTGATT-3′	5′-TTTTCTTGCTGCCAGTCTGGAC-3′
HPRT	5′-TGACCTTGATTTATTTTGCATACC-3′	5′-CGAGCAAGACGTTCAGTCCT-3′
BMP2	5′-AGACCTGTATCGCAGGCACT-3′	5′-CCTCCGTGGGGATAGAACTT-3′
BMP4	5′-CTTTACCGGCTTCAGTCTGG-3′	5′-GGGATGCTGCTGAGGTTAAA-3′
BMPR-IA	5′-GAAAAAGTGGCGGTGAAAGT-3′	5′-TAGAGCTGAGTCCAGGAACC-3′
BMPR-IB	5′-GCCAGCTGGTTCAGAGAGAC-3′	5′-CAGGACCCTGTCCCTTTGAT-3′
BMPRII	5′-TAGCACCTGCTATGGCCTTT-3′	5′-CTGAATTGAGGGAGGAGTGG-3′
VDR	5′-CTGACCCTGGAGACTTTGAC-3′	5′-TTCCTCTGCACTTCCTCATG-3′
CAMP	5′-GCAGTCACCAGAGGATTGTGAC-3′	5′-CACCGCTTCACCCAGCCC-3′
CYP24A1	5′-TGGCTTCAGGAGAAGGAAAA-3′	5'-ACCAGGGTGCCTGAGTGTAG-3′
CYP27B1	5′-GTTGCTATTGGCGGGAGTGGAC-3′	5′-GTGACACAGAGTGACCAGCGTAT-3
PRTN3	5′-AGGAGCTCAATGTCACCGT-3′	5′-ATGCCATCACAGATCCAGGG-3′
NE	5′-CGTGCAGCGCATCTTCGAA-3′	5′-ACGTTGGCGTTGATGGTGG-3′
TREML2	5′-TCTATGGTTTTGGAAGAAGAGACA-3′	5′-TCCAGGTGGGTCACGTGTAG-3′

Flow cytometry analysis

Cells were stained 20 min at 4°C with antibodies specifically recognizing CD34, CD38 (Becton Dickinson), BMPR1A (R&D system), and Vitamin D receptor (12550S, Cell Signalling) or a relevant isotype-matched control antibody.

Immunofluorescence staining

The cells were washed, spun onto slides, fixed with acetone, hydrated with cold 1% BSA in 1X PBS for 30 min, treated with formaldehyde 2% (Sigma) for 20 min, and then with methanol (Prolabo) for 10 min at room temperature. Next, the cells were permeabilized with 0.2% Triton X100 (Sigma) in 1X PBS for 10 min at 4 °C, washed with 1% BSA in 1X PBS and incubated in 3% BSA for 30 min. They were then sequentially incubated with the antibodies as follows: anti-VDR overnight at 4 °C (ab3508, Abcam), anti-rabbit-AF488 (Invitrogen) for 45 min at room temperature. All antibodies were diluted in 1% BSA and 0.1% Tween (Sigma) in 1X PBS. Nuclei were stained with DAPI. For nucleus/cytoplasmic specific staining analysis, cells were washed by PBS, fixed with paraformaldehyde 4% (Sigma) 20 min and were washed with PBS twice. Next, the cells were permeabilized with 0.3% Triton X100 (Sigma) and incubated in 3% BSA in 1X PBS for 30 min, washed with 1X PBS. They were then sequentially incubated with the antibodies as follows: anti-VDR MA1-710 (Invitrogen) overnight at 4 °C, anti-rat A11006 (Life Technology) for 45 min at room temperature. All antibodies were diluted in 1% BSA in 1X PBS. Nuclei were stained with Sytox deep red. Cells were transferred onto slides, dried, mounted in IF mounting media and coverslipped.

Immunofluorescence image assessment

The confocal immunofluorescence images of CD34+ bone marrow derived cells of healthy donor, HL-60 and KG1A cells were analyzed using Fiji software. The nuclear delineation was done using the default threshold plugin, the cytoplasmic delineation was done using Huang threshold plugin. The stain intensity was measured in the cytoplasmic or the nuclear region using the tools of region of interest manager. The micrograph images of different experimental condition were collected with the same settings.

Datasets

Normalized Reads Per Kilobase Million (RPKM) Illumina HumanHT-12 microarray-based gene expression profiles from 227 CD34/CD38 cell fractions from 78 AML patients were obtained from the GSE76008 AML dataset20. One hundred and sixty-one patients in the TCGA AML cohort had both RNA-seq and clinical data. RPKM values were log-transformed to the base two after adding a value of 1. Expression levels were extracted from the log-transformed data. A representative probe set with the highest average intensity was selected for each gene.

Statistics

Mean comparisons were performed with bilateral Mann-Whitney test (paired or unpaired as required) and correlations were determined with Pearson bilateral test, using GaphPadPrism software (San Diego, CA, US). We performed a matrix correlation between a set of variables using Spearman non-parametric bilateral test (GraphPad Prism), confidence interval of 95%. The graph represents the coefficient of correlation ρ, ranging from -1 (maximal negative) to +1 (maximal positive) illustrated by a scale of colors (R software, Corrplot package). Significant p-values are indicated by an asterisk (NS: $p>0.05$, *: $p \leq 0.05$, ** $p \leq 0.01$, ***: $p \leq 0.001$, ****: $p \leq 0.0001$).
